# *Pseudomonas viridiflava*: An internal outsider of the *Pseudomonas syringae* species complex

**DOI:** 10.1111/mpp.13133

**Published:** 2021-08-31

**Authors:** Savana M. Lipps, Deborah A. Samac

**Affiliations:** ^1^ Plant Pathology University of Minnesota Twin Cities St Paul MN USA; ^2^ USDA‐ARS PSRU St Paul MN USA

**Keywords:** pathogenicity island, pectate lyase, phase variant, *Pseudomonas viridiflava*, species delineation, type III secretion system

## Abstract

*Pseudomonas viridiflava* is a gram‐negative pseudomonad that is phylogenetically placed within the *Pseudomonas syringae* species complex. *P*. *viridiflava* has a wide host range and causes a variety of symptoms in different plant parts, including stems, leaves, and blossoms. Outside of its role as a pathogen, *P*. *viridiflava* also exists as an endophyte, epiphyte, and saprophyte. Increased reports of *P*. *viridiflava* causing disease on new hosts in recent years coincide with increased research on its genetic variability, virulence, phylogenetics, and phenotypes. There is high variation in its core genome, virulence factors, and phenotypic characteristics. The main virulence factors of this pathogen include the enzyme pectate lyase and virulence genes encoded within one or two pathogenicity islands. The delineation of *P*. *viridiflava* in the *P*. *syringae* complex has been investigated using several molecular approaches. *P*. *viridiflava* comprises its own species, within the complex. While seemingly an outsider to the complex as a whole due to differences in the core genome and virulence genes, low average nucleotide identity to other of *P*. *syringae* complex members, and some phenotypic traits, it remains as part of the complex. Defining phylogenetic, phenotypic, and genomic characteristics of *P*. *viridiflava* in comparison to other *P*. *syringae* members is important to understanding this pathogen and for the development of disease resistance and management practices.

**Taxonomy:**

Kingdom Bacteria; Phylum Proteobacteria; Class *Gammaproteobacteria*; Family *Pseudomonadaceae*; Genus *Pseudomonas*; Species *Pseudomonas syringae* species complex, Genomospecies 6, Phylogroup 7 and 8.

**Microbiological properties:**

Gram‐negative, fluorescent, aerobic, motile, rod‐shaped, oxidase negative, arginine dihydrolase negative, levan production negative (or positive), potato rot positive (or negative), tobacco hypersensitivity positive.

**Genome:**

There are two complete genomes, five chromosome‐level genomes, and 1,540 genomes composed of multiple scaffolds of *P*. *viridiflava* available in the National Center for Biotechnology Information Genome database. The median total length of these assemblies is 5,975,050 bp, the median number of protein coding genes is 5,208, and the median G + C content is 59.3%.

**Disease symptoms:**

*P*. *viridiflava* causes a variety of disease symptoms, including spots, streaks, necrosis, rots, and more in above‐ and below‐ground plant parts on at least 50 hosts.

**Epidemiology:**

There have been several significant disease outbreaks on field and horticultural crops caused by *P*. *viridiflava* since the turn of the century. *P*. *viridiflava* has been reported as a pathogen, epiphyte, endophyte, and saprophyte. This species has been isolated from a variety of environmental sources, including asymptomatic wild plants, snow, epilithic biofilms, and icepacks.

## Graphical abstract

*Pseudomonas viridiflava* is a generalist that is characterized by its phenotypic plasticity, intraspecies genetic diversity, utilization of pectate lyase, and pathogenicity island variation.

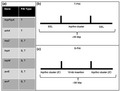

## 1 INTRODUCTION

*Pseudomonas viridiflava* is a species in the *Pseudomonas syringae* species complex, an amalgam of closely related pseudomonads that altogether comprises nine genomospecies, 13 phylogroups, and 60 pathovars with a vast host range (Berge et al., 2014; Gardan et al., 1999). Originally isolated in Switzerland from dwarf bean (*Phaseolus vulgaris*) with reddish‐brown lesions on the pods in 1930 (Billing, 1970), *P*. *viridiflava* has been shown to infect a range of both monocot and dicot hosts; it has been reported to cause disease in over 50 hosts (Table 1). Since 2000, there have been at least 13 outbreaks of diseases on annual plants caused by *P*. *viridiflava*; this makes up 18% of all outbreaks caused by the entire *P*. *syringae* species complex worldwide (Lamicchane et al., 2015). Diseases caused by *P*. *viridiflava* have been reported in countries such as Saudi Arabia, New Zealand, Italy, Hungary, Spain, and Greece, among others (Gonzáles et al., 2003; Morretti et al., 2005; Sarris et al., 2012; Taylor et al., 2011; Végh et al., 2012). In addition to its role as a crop pathogen, *P*. *viridiflava* also acts as an endophyte, epiphyte, and saprophyte in both agricultural and natural environments. Genomic and phenotypic characteristics of *P*. *viridiflava* make it an “internal outsider” within the *P*. *syringae* species complex. For example, while *P*. *viridiflava* is lumped within the *P*. *syringae* complex, it has distinct characteristics, including pectate lyase as a virulence factor, atypical pathogenicity islands, and phenotypic phase variation (Araki et al., 2006; Bartoli et al., 2014; Liao et al., 1988). Delineation of *P*. *viridiflava*, among other species within the *P*. *syringae* species complex, has been a major research focus in the past decade (Baltrus et al., 2017; Berge et al., 2014; Bull & Koike, 2015; Dillon, Thakur, et al., 2019). Genetic relationships have been investigated through techniques such as DNA–DNA hybridization, comparisons of 16S rRNA and housekeeping gene sequences, molecular fingerprinting, and more recently comparisons of whole‐genome sequences (Anzai et al., 2000; Berge et al., 2014; Dillon, Thakur, et al., 2019; Gardan et al., 1999). Another recent focus of *P*. *viridiflava* research is understanding the genetic and phenotypic variability within the species (Bartoli et al., 2014, 2015). Intraspecies phylogeny and pathogenicity island variability have been investigated (Araki et al., 2006, 2007; Bartoli et al., 2014). For phenotypic variability, research has focused on soft rotting potential and the two main colony phenotypes, transparent and mucoid, which exhibit varied pathogenic and antibiotic‐resistant phenotypes (Bartoli et al., 2014, 2015). This pathogen profile will highlight the research regarding current knowledge and advances in (1) delineating *P*. *viridiflava* in the *P*. *syringae* species complex, (2) lifestyle and epidemiology, (3) host range, (4) virulence, and (5) suggestions for future research on *P*. *viridiflava*.

**TABLE 1:** All plant hosts in which *Pseudomonas viridiflava* has been reported to cause disease in natural infections or in experimental conditions

Host/source	Symptoms	Reference
*Acanthus mollis*	Leaf blight	Sarris et al. (2012)
Alfalfa (*Medicago sativa*)	Stem blight, wilt and crown root rot	Heydari et al. (2014); Lipps et al. (2019)
Apple (*Malus domestica*)	Blossom blight	Alimi et al. (2011)
Apricot (*Prunus armeniaca*)	Bacterial canker on trunk	Parisi et al. (2019)
*Arabidopsis thaliana*	Leaf necrosis	Jakob et al. (2002)
Artichoke (*Cynara scolymus*)	Leaf necrosis	Sarris et al. (2012)
Basil (*Ocimum basilicum*)	Leaf necrosis	Little et al. (1994); Minuto et al. (2008)
Blite (*Suaeda maritima*)	Leaf spot	Goumans and Chatzaki (1998)
Broccoli (*Brassica oleracea* var. *italica*)	Head rot	Canaday et al. (1991)
Cabbage (*Brassica oleracea* var. *capitata*)	Leaf spot	Askoy et al. (2017)
*Calendula officinalis*	Leaf spot	Moretti et al. (2012)
Calla lily (*Zantedeschia aethiopica*)	Leaf spot	Basavand et al. (2019)
Canola (*Brassica napus*)	Leaf spot	Myung et al. (2010)
Carrot (*Daucus carota*)	Seed contamination, postharvest soft rot	Almeida et al. (2013); Godfrey and Marshall (2002)
Cauliflower (*Brassica oleracea* var. *botrytis*)	Leaf rot	Wilkie et al. (1973)
Celery (*Apium graveolens*)	Leaf blight	Sarris et al. (2012)
Cherry (*Prunus avium)*	Not specified	Harzallah et al. (2004); Morris et al. (2019); Ruinelli et al. (2017)
Chinese gooseberry (*Actinidia chinensis*)	Blossom blight	Wilkie et al. (1973)
Chrysanthemum	Stem rot, leaf necrosis	Goumans and Chatzaki (1998)
Citrus (*Citrus macrophylla, Citrus sinensis, Citrus aurantium*)	Citrus blast of leaf, black pit disease of fruit	Beiki et al. (2016)
Clover (*Trifolium pratense*)	Not specified	Morris et al. (2019)
Common water plantain (*Alismo plantago‐aquatica*)	Leaf spot	Basavand and Khodaygan (2020)
Cowpea (*Vigna unguiculata*)	Not specified	Morris et al. (2019)
Cucumber (*Cucumis sativus*)	Leaf spot	Wilkie et al. (1973)
Dwarf bean (*Phaseolus vulgaris*)	Reddish‐brown lesions on the pods	Burkholder (1930)
Eggplant (*Solanum melongena*)	Leaf spot	Goumans and Chatzaki (1998)
Favabean (*Vicia faba*)	Not specified	Morris et al. (2019)
Garlic (*Allium sativum*)	Leaf streak	Pérez Faggiani et al. (2004)
Geranium (*Pelargonium*)	Not specified	Morris et al. (2019)
Golden currant (*Ribes aureum*)	Not specified	Baltrus et al. (2017)
Grape (*Vinus vinifera*)	Panicle rot	Wilkie et al. (1973)
*Hebe* spp.	Leaf spot	González and Rodicio (2006)
Hellebore (*Helleborus* x *hybridus*)	Leaf spot, petal and stem lesions	Taylor et al. (2011)
Kiwifruit (*Actinidia deliciosa*)	Blossom blight	Everett and Henshall (1994)
Lettuce (*Latuca sativa*)	Leaf necrosis	Gonzales et al. (2003); Goumans and Chatzaki (1998)
Lupin (*Lupinus angustifolius*)	Leaf spot	Wilkie (1973)
Melon (*Cucumis melo*)	Leaf spot, leaf necrosis	Goumans and Chatzaki (1998)
Mexican heather (*Cuphea hyssopifolia*)	Leaf spot	Albu et al. (2018)
Mustard (*Brassica rapa*)	Not specified	Morris et al. (2019); Sayama et al. (2001)
Onion (*Allium sepa*)	Bacterial streak, bulb rot	Gitatis et al. (1997)
Parsnip (*Pastinaca sativa*)	Petiole soft rot	Hunter and Cigna (1981)
Passionfruit (*Passiflora edulis*)	Leaf blotch	Wilkie et al. (1973)
Pea (*Pisum sativum*)	Wet rot of leaves, stipules, and stems	Wilkie et al. (1973)
Peach (*Prunus persica*)	Not specified	Harzallah et al. (2004); Morris et al. (2019)
Plum (*Prunus domestica*)	Bacterial canker on trunk	Bophela et al. (2020)
Poppy (*Papaver somniferum*)	Stem rot	Wilkie et al. (1973)
Pumpkin (*Cucurbita maxima*)	Leaf spot	Wilkie et al. (1973)
Red‐leaved chicory (*Cichoriu intybus*)	Leaf spot	Caruso and Catara (1996)
*Saposhnikovia divaricata*	Leaf blight	Wang et al. (2015)
Sorghum (*Sorghum bicolor*)	Not specified	Morris et al. (2019)
Soybean (*Glycine max*)	Dark‐reddish spot	Gonzales et al. (2012)
Sunflower (*Helianthus annus*)	Not specified	Morris et al. (2019)
Sweet crab apple (*Malus coronaria*)	Shoot blight	Choi et al. (2020)
Tomato (*Solanum lycopersicum*)	Internal stem rot, bacterial blight, pith necrosis	Jones et al. (1984); Saygili et al. (2008); Wilkie et al. (1973)

## 2 DELINEATION WITHIN THE *P. syringae* SPECIES COMPLEX

### 2.1 Taxonomy

A goal of recent *P*. *syringae* species research is to clarify the taxonomic delineation of associated species and pathovars, including *P*. *viridiflava*. Currently, *P*. *syringae* is referred to as a species complex, defined as a cluster of related monophyletic groups based on historical trends in bacterial classification initially based on phenotypes and progressively based on genotypes including DNA–DNA hybridization and phylogenetic analysis of housekeeping genes sequences (Berge et al., 2014). In the past, the pseudomonads were differentiated using the LOPAT profile test (levan production, oxidase production, pectinolytic activity, arginine dihydrolase production, and tobacco hypersensitivity) and DNA–DNA hybridization (Gardan et al., 1999; Palleroni, 1984). Subsequently, 16S rRNA gene sequences were used to delineate associated *P*. *syringae* at the species level, which resulted in *P*. *viridiflava* being recognized as a part of the *P*. *syringae* species complex (Anzai et al., 2000). Because the *P*. *syringae* species complex is large and diverse, improved methods for detecting species diversity and grouping similar organisms were developed. Sequences from housekeeping genes, *gyrB* and *rpoD*, as well as the 16S rRNA gene, were originally used to assemble a phylogeny of members of the *P*. *syringae* species complex and delineate *P*. *viridiflava*, among others (Yamamoto et al., 2000). Since then, other housekeeping genes such as *gapA*, *cts*, *rpoB*, and *purA* and more have been sequenced and used to create phylogenetic trees either with a single gene or in multilocus sequence analysis (MLSA) using *P*. *viridiflava* isolates (Berge et al., 2014; Goss et al., 2005; Parisi et al., 2019; Parkinson et al., 2011; Sarris et al., 2012). It has been suggested that the *cts*, *gapA*, or *rpoD* housekeeping genes alone are sufficient to place a *P*. *viridiflava* isolate into its phylogroup in the *P*. *syringae* complex (Berge et al., 2014; Parkinson et al., 2011). In identification of *P*. *viridiflava* and other *P*. *syringae* species, Bull and Koike (2015) created a framework for determining the aetiology of bacterial plant pathogens at the intraspecies level, which involves molecular fingerprinting known as rep‐PCR (repetitive extragenic sequence palindromic‐polymerase chain reaction), housekeeping gene sequencing for MLSA, and pathogenicity testing.

### 2.2 Intraspecies phylogeny

Currently, the *P. syringae* species complex comprises nine genomospecies, 13 phylogroups, and 64 pathovars. A genomospecies within the *P*. *syringae* complex is defined by DNA–DNA hybridization ability; through this method, *P*. *viridiflava* was determined to be a distinct species from other members of *P*. *syringae* sensu lato (Gardan et al., 1999). *P*. *viridiflava* strains do not hybridize with other species in the *P*. *syringae* complex (Gardan et al., 1999). In a study by Gomila et al. (2017) including type strain ICMP 2848, *P*. *viridiflava* had an average nucleotide identity of less than 97% compared to other members of the species complex, which is below the accepted threshold for species separation (Goss et al., 2005). Phylogroup delineations were determined by Berge et al. (2014) using the four‐gene MLSA classification schema developed by Hwang et al. (2005). The term “pathovar” is a naming convention used for granular subdivision within phylogroups to associate pathogenic strains with information about the host of origin, mainly in description of pathotype strains. *P*. *viridiflava* strains are represented in genomospecies 6, which contains phylogroups 7 (PG7) and 8 (PG8) (Berge et al., 2014; Gardan et al., 1999). PG7 is further subdivided into PG7a, represented by pathotype strain *P*. *syringae* pv. *primulae* LMG2252 and PG7b represented by pathotype strain *P*. *viridiflava* FMU107; PG8 is represented by pathotype strains *P*. *viridiflava* LMG2352 and *P*. *syringae* pv. *ribocola* LMG‐2276 (Berge et al., 2014; Bull & Koike, 2015; Gardan et al., 1999). The majority of named “*P*. *viridiflava*” strains group within PG7a and are typically isolated from a variety of environmental sources (Berge et al., 2014). Some key features of PG7 are the soft‐rotting capability on potato tubers, phenotypic phase variation, and the presence of a noncanonical type III secretion system (T3SS) (Bartoli et al., 2014). PG8 shares the key features described for PG7, but it is differentiated by the production of a toxin in bioassays with *Geotricum candidum* (Berge et al., 2014). *P*. *viridiflava* exhibits high intraspecific genetic variation. In the paramount study by Bartoli et al. (2014) assessing the intraspecific variability of *P*. *viridiflava*, the genetic diversity of strains was characterized per the structure and sequences of the pathogenicity islands (PAIs), critical portions of the genome for virulence. Not only did strains contain divergent types of PAIs, both single‐partite (S‐PAI) and tripartite (T‐PAI), but several strains of the S‐PAI type contained an exchangeable effector locus that was not present within other S‐PAI types, but present in all T‐PAI types (Bartoli et al., 2014). *P*. *viridiflava* strains contain higher diversity in PAI structure compared to *P*. *syringae* sensu stricto strains, which typically only contain one type of canonical T‐PAI (Dillon, Thakur, et al., 2019). Genetic diversity was also previously demonstrated in a population of *P*. *viridiflava* strains isolated from *Arabidopsis thaliana* from various geographic locations (Goss et al., 2005). There was substantial variation in the five genomic fragments examined, with an average of 33.4% synonymous site nucleotide divergence between two clades defined within the population, and 9.3% synonymous site nucleotide divergence within a single clade. This variation was not correlated with differences in geographic location of isolates. In another study, the hypothesis that intraspecific genetic variation of *P*. *viridiflava* is not due to host‐specific adaptation was supported, given by divergent clade groupings in which strains isolated from different hosts grouped together within clades (Sarris et al., 2012). A recent study on the intraspecies genetic diversity within the *P*. *syringae* complex reveals that while recombination and horizontal gene transfer (HGT) occur frequently for most strains in the complex regardless of phylogroup delineation, nonagricultural, environmental strains from PG13, PG7, and PG11 experience highest rates of HGT in that order (Dillon, Thakur et al., 2019). Environmental strains, as opposed to agricultural strains, may contain more HGT‐obtained loci from other species due to increased opportunities to interact with various microbial species (Dillon, Thakur et al., 2019). High rates of HGT in PG7 strains, as well as strains from other phylogroups, may contribute to its intraspecific diversity. The evolutionary potential of *P*. *viridiflava* due to high rates of HGT and occupation of varied niches may contribute to the intraspecific diversity.

Although MLSA and molecular fingerprinting techniques, including rep‐PCR, have been useful tools for exploring intraspecies diversity in the past, the increase in affordability and accessibility of whole‐genome sequencing will soon be the gold standard for delineating *P*. *viridiflava* and other members within the complex. In the past several years, genome sequences of *P*. *viridiflava* have been generated and used for analyses and taxonomic classification (Dillon, Thakur, et al., 2019; Gomila et al., 2017; Ruinelli et al., 2017; Samad et al., 2017; Thakur et al., 2016). There are over 1,500 *P*. *viridiflava* draft genome assemblies available in the National Center for Biotechnology Information Genome Database. The first complete assembly of a genome sequence for *P*. *viridiflava* was of strain CFBP‐1590, a strain from PG7 isolated from diseased cherry in France (Ruinelli et al., 2017); this genome is 6.09 Mb, has a G + C content of 59.2%, and contains 5,283 protein‐coding sequences (accession no. LT855380). The second complete genome of *P*. *viridiflava* is that of strain U625 in PG7 isolated from alfalfa (*Medicago sativa*) with bacterial stem blight disease. This strain has a genome of 5.997 Mb, G + C content of 59.2%, and contains approximately 5,468 protein‐coding sequences (accession no. CP074412). Recently, an impressive whole‐genome sequencing and evolutionary analysis of 391 agricultural and environmental strains of *P*. *syringae*, including four *P*. *viridiflava* strains in PG7, revealed that there are primary and secondary phylogroups within the complex based on genomic relatedness (Dillon, Thakur, et al., 2019). While most strains (PG1, PG2, PG3, PG4, PG5, PG6, PG10) group into the primary group, *P*. *viridiflava* strains of PG7 grouped within the secondary group. PG8 isolates of *P*. *viridiflava* were not included in this analysis, but probably can be considered members of the secondary phylogroup due to genetic and phenotypic similarities to PG7 strains.

[Correction added on 23 September 2021, after first online publication: The type strain in the cited study by Gomila et al. (2017) in Section 2.2 has been corrected in this version.]

## 3 EPIDEMIOLOGY AND LIFESTYLE

*P. viridiflava* is widely distributed and plays various roles in the environment. This species has been reported as an epiphyte, endophyte, saprophyte, and pathogen on a variety of agricultural and wild plant hosts (Bartoli et al., 2014; Bordjiba & Pruner, 1989; Samad et al., 2017). *P*. *viridiflava* has also been commonly isolated from nonplant environmental reservoirs, such as snowpack, rain, epilithic biofilms, and lake water (Bartoli et al., 2014; Pietsch et al., 2017). Like *P*. *syringae*, some *P*. *viridiflava* strains have ice nucleation capabilities, which supports their potential relevance in nonagricultural environments such as the water cycle. Studies show *P*. *viridiflava* isolates having ice nucleation capabilities in roughly 33%–45% of strains tested (Berge et al., 2014; Pietsch et al., 2017). Although widespread, its broad distribution does not seem to follow a particular geographical pattern or structure (Sarris et al., 2012). Because members of the *P*. *syringae* species complex, including *P*. *viridiflava*, are seemingly ubiquitous throughout various environments yet diverse in population structure, it is conceivable to place *P*. *viridiflava* into the ecotype model by Cohan (2002), which emphasizes the role of recurrent selective sweeps in defining the niche of distinct populations of bacteria (Baltrus et al., 2017). Therefore, the existence of epiphytic, endophytic, and pathogenic states could be considered ecotypes of *P*. *viridiflava* shaped by certain environmental conditions and selection pressures. Goss et al. (2005) hypothesize that because *P*. *viridiflava* is often deemed a weak or opportunistic pathogen, it could experience selection pressure in its epiphytic phase that the pathogenic ecotypes do not. *P*. *viridiflava* has demonstrated various roles in the microbial community as an epiphyte and endophyte. Some *P*. *viridiflava* isolates have been shown to produce a family of antimycotics, called ecomycins, that have significant bioactivity against both human and plant‐pathogenic fungi (Miller et al., 1998). Presumably, this capability could be important to establishing as a plant epiphyte or pathogen when encountering fungal competitors. *P*. *viridiflava* has also been identified as an endophyte of weeds with the capability of herbicidal activity (Samad et al., 2017). The *P*. *viridiflava* strain CDRTc14, originally isolated as an endophyte in a vineyard in Australia, significantly inhibited seed germination and root growth of the weed *Lepidium draba* in greenhouse conditions (Samad et al., 2016). The CDRTc14 genome contained abiotic stress tolerance genes, such as genes for heavy metal and herbicide resistance, but it did not contain a complete pathogenicity island or pathogenicity phenotype typical of pathogenic *P*. *viridiflava* strains. The ability of *P*. *viridiflava* to act as an endophyte, saprophyte, and pathogen supports the idea that *P*. *viridiflava*, like many other members of the complex, is a generalist rather than a specialist. Its ability to infect a wide range of hosts corroborates its validity as a generalist pathogen (Goss et al., 2005; Lamichhane et al., 2015).

*P. viridiflava* is responsible for 13 economically relevant disease outbreaks on annual plants since 2000 (Lamichhane et al., 2015) and has been reported to cause disease on over 50 hosts since its discovery (Table 1). There are various sources of inoculum for *P*. *viridiflava* infection. *P*. *viridiflava* has been detected in a range of environmental sources, including epilithic biofilms, rain, irrigation water, and litter; presumably, these could all be sources of inoculum to relevant crop hosts. *P*. *viridiflava* inoculum can also come from contaminated seeds **(**Almeida et al., 2013; Yildiz et al., 2004). Epiphytic populations of *P*. *viridiflava* may also be a source of inoculum, as selection pressure may change epiphytic populations to pathogens under certain conditions. Because *P*. *viridiflava* is detected in water sources, it is conceivable that, like *P*. *syringae*, it can be disseminated by aerosols, rain, and wind. *P*. *viridiflava* can survive on plant surfaces or enter the host through stomata, hydathodes, or wounds. Overall, disease is most likely to occur when the host experiences stress such as low temperatures, high levels of rainfall, high humidity, or prior wounding (Jakob et al., 2002; Lamicchane et al., 2015). Ice nucleating properties of *P*. *viridiflava* may be beneficial to create frost wounds on the host, which can serve as an entry point for the bacteria (Lindow et al., 1982; Varvaro & Fabi, 1992).

An extensive study was performed on the epidemiology of *P*. *viridiflava* causing kiwifruit blossom blight over two decades ago. Interestingly, data from this study showed that weather variables (air temperature, surface wetness, rainfall, and relative humidity) did not seem to affect development of disease while timing of the infection before the budding phase was critical for disease (Everett & Henshall, 1994). Since then, there have not been any studies solely dedicated to understanding the epidemiology of *P*. *viridiflava* in causing disease on other specific hosts. Although there may be evidence that climate variables do not affect disease development in the case of kiwifruit blossom blight, *P*. *viridiflava* infects such a wide range of hosts that the role of climate variables needs to be studied in other host systems. There is a need for more epidemiological (disease progression) studies of *P*. *viridiflava* isolates causing disease on its wide range of hosts.

Another aspect to consider regarding epidemiology and symptom expression by *P*. *viridiflava* is its potential for synergism with other microbes. There have been several reports of *P*. *viridiflava* causing disease in synergy with other microbes. For example, *P*. *viridiflava* causes tomato pith necrosis either by itself or in association with seven other *Pseudomonas* species. Interestingly, it was found that disease severity is greater in co‐infections of *P*. *viridiflava* with one or more of the other species (Lamichhane & Venturi, 2015). Also, bacterial strains across different species including *Pectobacterium carotovorum, Pseudomonas marginalis*, *Pseudomonas* *fluorescens*, and *P*. *viridiflava* have been reported to cause broccoli head rot, of which symptoms are ultimately attributed to this bacterial complex (Canaday et al., 1991). Most recently, *P*. *viridiflava* was reported to cause bacterial stem blight disease of alfalfa along with *P*. *syringae* PG2 strains (Lipps et al., 2019). The mechanism of synergy is currently unknown in this system. Overall, the synergistic potential of *P*. *viridiflava* with other microbes may be a factor in its ability to cause disease in certain situations.

## 4 HOST RANGE

*P. viridiflava* has a wide natural and experimental host range. This species has also been isolated as an endophyte and epiphyte of wild plants as well as from various environmental sources. Here, the currently known host range of *P*. *viridiflava* as a pathogen is summarized, including data from natural hosts and explicit host range studies (Table 1).

There is evidence for variability in the capability of *P*. *viridiflava* to cause disease on different hosts depending on strain. For example, a *P*. *viridiflava* isolate was reported to cause disease on soybean (Gonzáles et al., 2012), but in the host range test by Morris et al. (2019) the *P*. *viridiflava* isolate used did not cause disease on soybean. Also, a recent report of *P*. *viridiflava* causing disease on plum (*Prunus domestica*) cultivars Sapphire and Songold in South Africa (Bophela et al., 2020) contradicts the result of a “no disease” outcome in a host range test with plum cultivar Marina GF8‐1 by Morris et al. (2019); this may be an example of cultivar‐specific resistance. These examples of varied capacity for causing disease are similar to the observations that different isolates within the same species of *P*. *syringae* can have variable capabilities on different hosts (Morris et al., 2019). The variable capacity of *P*. *viridiflava*‐related isolates to cause disease in different hosts warrants testing the host range potential of a diverse array of *P*. *viridiflava* isolates under varied conditions to fully understand the factors of pathogenic potential of this species. In addition to understanding how environmental conditions contribute to disease‐induction variability, it is important to understand the role of virulence genes and genomic regions that may contribute to the pathogenic success of *P*. *viridiflava*. For example, findings of Bartoli et al. (2014) suggest that the presence or absence of the virulence gene, *avrE*, is crucial to the virulence of this pathogen. *P*. *viridiflava* is considered a generalist pathogen due to its demonstrated ability to induce disease on a wide variety of hosts. The generalist style of *P*. *viridiflava* may be partially explained by its utilization of pectate lyase, a nonhost‐specific enzyme used to degrade pectin, as a major virulence factor, as well as a simplified pathogenicity island lacking effectors targeted toward specific hosts, both of which are further explained in the next section.

## 5 VIRULENCE

### 5.1 Soft rot/pectate lyase

Secretion of pectate lyase to degrade pectin in plant cell walls is one of the main virulence strategies of *P*. *viridiflava*. Pectate lyase depolymerizes pectin and other polygalacturonates. The production of pectate lyase via the *pel* gene is responsible for the pectinolytic activity of *P*. *viridiflava* that results in soft rot and macerated plant tissue (Liao, 1991; Liao et al., 1988). Mutant strains with a defective *pel* gene resulted in no leaf maceration after infection on *Arabidopsis* (Jakob et al., 2007). Pectate lyase activity has been shown to differ based on the type of PAI of the bacterial strain; single‐PAI isolates exhibited twofold higher enzyme activity than tripartite‐PAI isolates on *Arabidopsis*, even though the *pel* gene is encoded outside of the PAI (Jakob et al., 2007). The production of pectate lyase may be considered a significant biological difference between *P*. *viridiflava* and other members of the *P*. *syringae* species complex. Although the soft rotting phenotype is unique to *P*. *viridiflava* within the *P*. *syringae* complex, a phenotypic study showed that 8% of sampled *P*. *viridiflava* strains were not able to induce soft rot on potato tubers, therefore soft rot may be used as a general descriptor for the species, but not a diagnostic trait (Bartoli et al., 2014).

### 5.2 Phase variation and mutability

**FIGURE 1:**
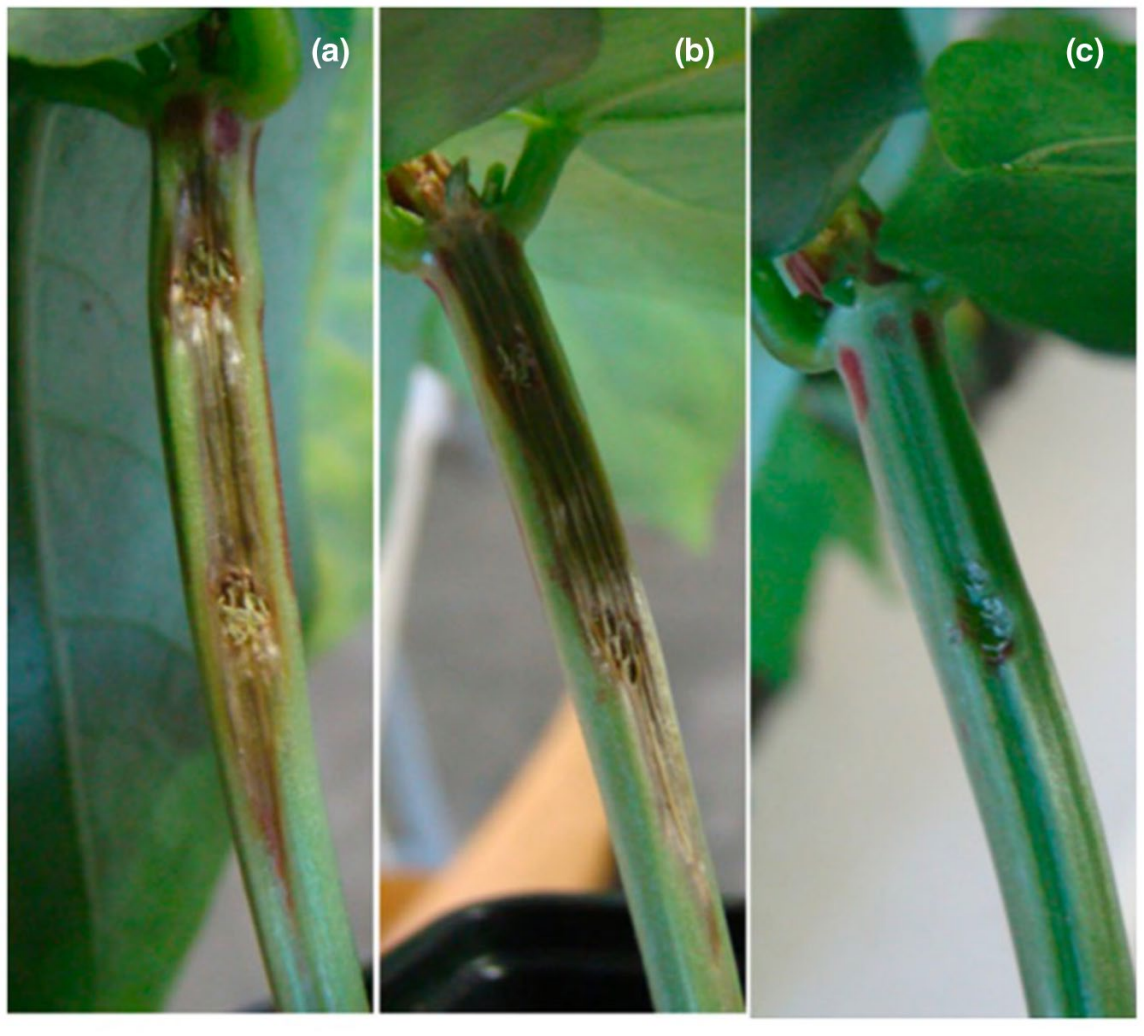
*Pseudomonas viridiflava* phase variant disease phenotypes. This figure from Bartoli et al. (2015) depicts the disease symptoms on bean (*Phaseolus vulgaris* ‘Pinto’) induced by the different variant clones of a wild‐type strain of *P*. *viridiflava* at 7 days postinoculation. (a) Disease phenotype of the wild‐type isolate. (b) Mucoid variant clone of the wild‐type isolate disease phenotype. (c) Disease phenotype of the transparent variant clone of the wild‐type isolate. Plants were point inoculated using bacterial suspensions at 10^8^ cfu/ml

An important discovery regarding *P*. *viridiflava* is the phenotypic plasticity of pathogenicity‐related traits. Historically, “levan‐production negative” was a characteristic of the typical LOPAT profile of *P*. *viridiflava*. However, yellow mucoid, levan‐positive bacterial colonies originally isolated from bean, kiwifruit, and lettuce were identified as an atypical form of *P. viridiflava* (Gonzáles et al., 2003). More recent discoveries of levan‐production positive *P*. *viridiflava* isolates, which also display yellow, mucoid growth on King's B medium, are evidence for phase variation within the species; in fact, 56% of *P*. *viridiflava* strains tested in a study by Bartoli et al. (2014) were levan‐production positive. Thus, the current knowledge of *P*. *viridiflava* phenotypic variability is that there are two phase variants of isolates: a yellow, mucoid, levan‐positive variant and a transparent, flat, levan‐negative variant. Interestingly, isolates can switch between the variant phenotypes, and the variants correlate with pathogenic potential. In a pathogenicity study of 11 mucoid strains and 11 transparent strains stably cloned from the same original 11 isolates, the mucoid variant could induce soft rot on potato tubers, while the nonmucoid variant could not (Bartoli et al., 2014). Also, wild‐type (defined as whichever of the two variants naturally occurred in original isolate) and mucoid variant isolates were able to induce disease on bean stems (Figure 1), while the transparent variant could not (Bartoli et al., 2015). Although phase variation in *P*. *viridiflava* could be linked to pathogenic potential, there may be other advantages to possessing this type of plasticity. The presence of exopolysaccharide could increase bacterial tolerance to plant defences, or the pectinolytic capability of the mucoid strains could be important to bacterial colonization via release of sugars (Bartoli et al., 2014). More recently, it was reported that the transparent variant has a mutator phenotype and general antibiotic resistance in additional to low pathogenic potential on bean (Bartoli et al., 2015). Conversely, the mucoid variant did not show mutability or antibiotic resistance potential but did effectively cause disease in bean. Though *P*. *viridiflava* strains are probably plastic in their mucoid and nonmucoid phenotypes, the genetics underlying this phase switch are currently unknown and may be of interest for future research.

### 5.3 Pathogenicity islands, associated virulence genes, and effectors

**FIGURE 2:**
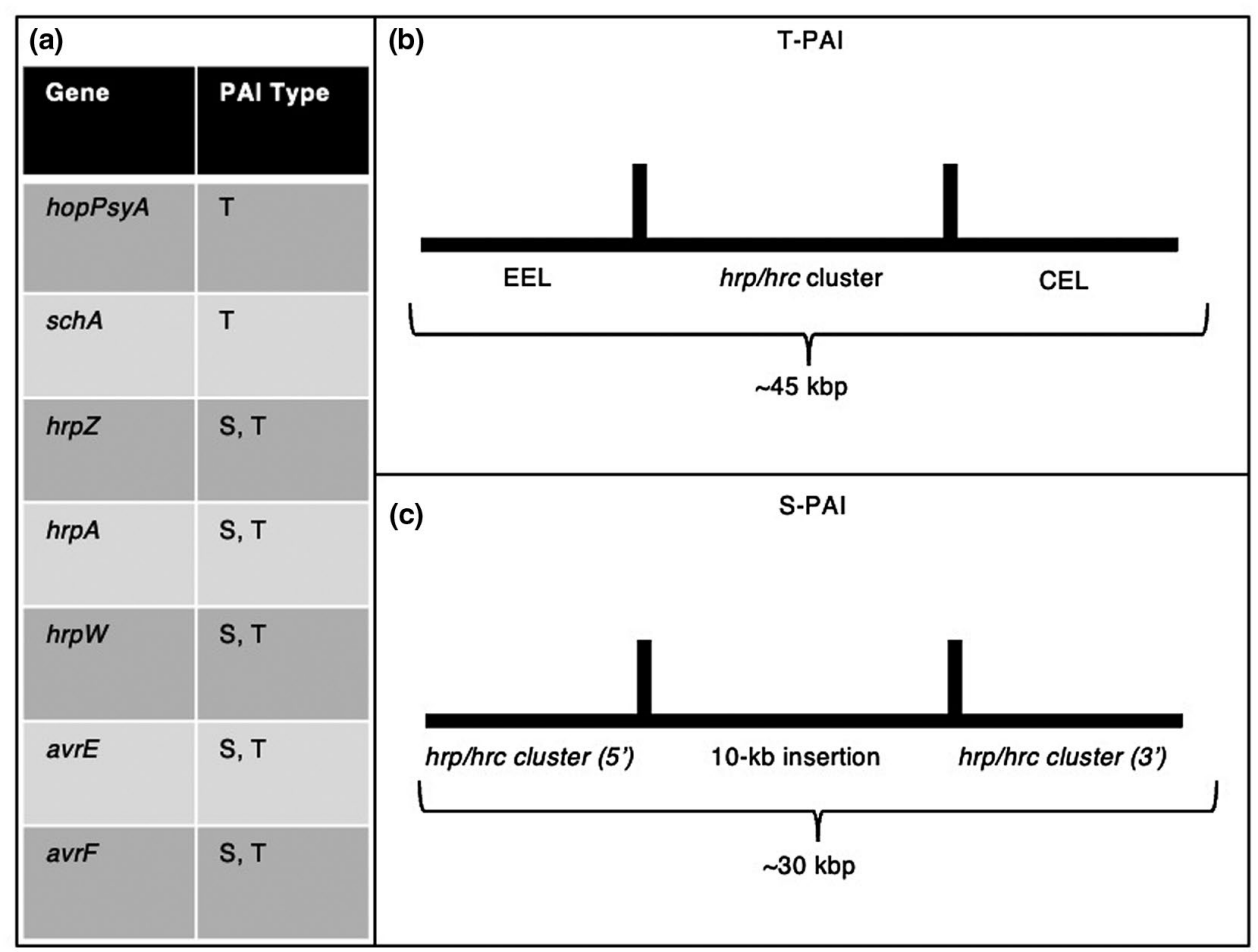
The pathogenicity island variants of *Pseudomonas viridiflava*, single‐partite (S‐PAI) and tripartite (T‐PAI). [Adapted from Araki et al. (2007).] The virulence genes and structures found within the two commonly found pathogenicity island variants in *P*. *viridiflava* from Araki et al. (2007). (a) List of virulence genes found in either S‐PAI (S) or T‐PAI (T). (b) Depiction of structure of T‐PAI including exchangeable effector locus (EEL), *hrp/hrc cluster*, and conservative effector locus (CEL). (c) Depiction of the structure of typical S‐PAI including the *hrp/hrc cluster* with an insertion

In the *P*. *syringae* complex and commonly in gram‐negative bacteria, virulence factors such as the type III secretion system (T3SS) and associated effectors are arranged in a cluster known as a pathogenicity island (PAI). In the early 2000s, Araki et al. (2006, 2007) contributed significantly to the understanding of the genetic basis of pathogenicity of *P*. *viridiflava*, specifically regarding PAIs, using isolates from *Arabidopsis*. There are two main forms of PAIs in *P*. *viridiflava* that exist as a presence/absence polymorphism in individual strains. The most recent common ancestor of the two PAI types predates the divergence of *P*. *viridiflava* from other *Pseudomonas* species. This serves as evidence that the two PAI types could not have originated from a recent HGT, or a recent duplication event; rather, there is a deeper evolutionary history of PAI development in this species (Araki et al., 2006). The two forms, a single pathogenicity island (S‐PAI) and a tripartite pathogenicity island (T‐PAI), differ in structure and phenotype. The T‐PAI contains three components: the *hrp*/*hrc* gene cluster, the 5′ effector locus or the exchangeable effector locus (EEL), and the 3′ effector locus or the conserved effector locus (CEL); the T‐PAI variant region is typically c.45 kb (Araki et al., 2006, 2007). The S‐PAI differs in that it only contains one of the components of the T‐PAI, the *hrp/hrc* cluster, as well as a 10 kb insertion; the S‐PAI variant region is typically c.30 kb (Araki et al., 2006, 2007). In Araki et al. (2007), S‐PAI‐associated virulence genes include *avrE* (avirulence gene), *avrF* (putative *avrE* chaperone), and *hrpA*, *hrpZ*, and *hrpW* (type III secreted proteins). In the same study, T‐PAI associated genes included those of S‐PAI as well as *hopPsyA* (avirulence gene) and *shcA* (putative *hopPsyA* chaperone) (Araki et al., 2007; Figure 2). An association between PAI type and host‐specific virulence was also noted; S‐PAI variant isolates were found to cause disease more rapidly on *Arabidopsis*, while the T‐PAI variant isolates were faster in causing a hypersensitive response (HR) in tobacco (Araki et al., 2006). In a study of 286 *P*. *viridiflava* isolates from around the world, 10% contained a T‐PAI and the other 90% contained an S‐PAI; in both cases, each isolate contained a single type of PAI (Araki et al., 2006). Thus, the majority of *P*. *viridiflava* isolates examined harboured a S‐PAI.

Since the work of Araki et al. (2006, 2007), the previous understanding that S‐PAI and T‐PAI do not share a common EEL region has shifted. In a study of environmental *P*. *viridiflava* isolates, a genomic region resembling an EEL was detected in S‐PAI strains, probably from a recombination between the two types of PAI (Bartoli et al., 2014). This amends the previous understanding that only two distinctly different PAIs, one containing an EEL and CEL and one without either, exist in *P*. *viridiflava*. Additionally, although it was previously accepted that the two different PAIs were associated with varied virulence phenotypes, recent research showed that the two PAI configurations are not correlated with pathogenicity or soft rotting capability; instead, it was found that the only gene linked with pathogenicity was the presence or absence of the *avrE* effector on the PAI (Bartoli et al., 2014). As sampling and sequencing of *P*. *viridiflava* increases, it is possible that there will be more isolates with variable PAIs due to recombination than have previously been discovered.

**FIGURE 3:**
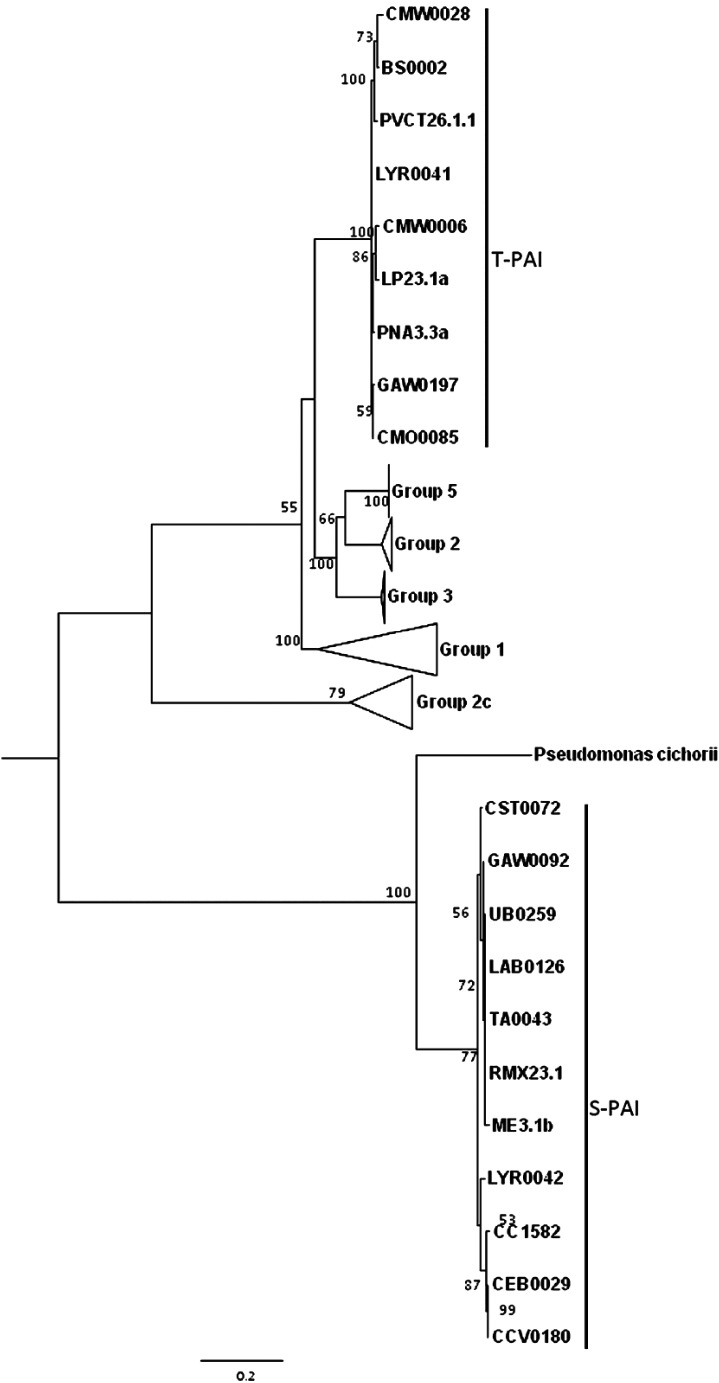
Phylogenetic tree of *Pseudomonas viridiflava* isolates constructed with pathogenicity island (PAI)‐associated gene *hrcC*. This figure from Bartoli et al. (2014) depicts the grouping of *P*. *viridiflava* isolates based on their type of pathogenicity island, either single‐partite (S‐PAI) or tripartite (T‐PAI), and their relationship to other phylogroups of the *P*. *syringae* species complex

To infer evolutionary history, Bartoli et al. (2014) constructed a phylogeny with the *hrcC* gene present in both PAI types and found that the T‐PAI strains grouped closest with PG5, PG2, and PG3 of the *P*. *syringae* complex, whereas the S‐PAI strains formed a group more closely related to *Pseudomonas* *cichorii* (Figure 3). This corroborates the findings in Araki et al. (2007) that the PAI types have a deep and divergent evolutionary history. It was also found that regardless of PAI type, there were two genes, coding for lipoprotein and monooxygenase, that were present in nearly all strains that were analysed by Bartoli et al. (2014). These genes are present in the EEL of the T‐PAI and in a region resembling an EEL, yet lacking effectors, in S‐PAI types. Phylogenetic analysis of these two genes showed that they grouped in accordance with their PAI type (Bartoli et al., 2014). Finally, in Bartoli et al. (2014) PG7 strains contained either an S‐PAI or T‐PAI type, while PG8 strains contained only the T‐PAI type. This finding led to the hypothesis that the T‐PAI in PG7 strains was probably acquired later in its evolutionary history, which was supported by the placement PG8 strains at the root of the phylogenetic tree of PG7 and PG8 strains constructed with four‐gene MLSA. In the context of all PGs within the *P*. *syringae* complex based on four‐gene MLSA (Berge et al., 2014), PG7 and PG8 group more proximally to PG11 (*P*. *cichorii*), which is closest to the root of the tree, than most other phylogroups. A distinct feature of *P*. *cichorii* is its oxidase‐positive phenotype. *P*. *viridiflava* does not have an oxidase‐positive phenotype, but the cytochrome c oxidase operon, responsible for this phenotype in *P*. *cichorii*, was found in two strains in PG7, but not in any other phylogroup (Berge et al., 2014). The shared S‐PAI and cytochrome c oxidase operon between *P*. *cichorii* and some PG7 strains is corroborative of their evolutionary history.

Recently, the phylogenetic distribution of the T3SS in *P*. *viridiflava* was studied. Four types of T3SS were detected in *P*. *viridiflava* strains: canonical T‐PAI, alternate T‐PAI (which acts as a replacement for the canonical T‐PAI), S‐PAI, and a *Rhizobium*‐like PAI, or R‐PAI, which differs from other PAIs by the splitting of the *hrcC* gene (Dillon, Thakur, et al., 2019; Gazi et al., 2012). Interestingly, all *P*. *viridiflava* isolates containing a S‐PAI T3SS also contained a R‐PAI T3SS. Likewise, the R‐PAI was always detected in tandem with at least one other type of T3SS across all strains tested in *P*. *viridiflava* and *P*. *syringae* generally. Even though the S‐PAI variant lacks some features of the canonical T3SS (i.e., the EEL and CEL), Dillon, Thakur, et al. (2019) hypothesized that these strains would be successful in effector delivery into some plant hosts. Further research by Dillon, Almeida, et al. (2019) suggests that there are fewer T3SS effectors present in the secondary phylogroups of *P*. *syringae*, which include *P*. *viridiflava* (PG7 and PG8), compared to primary phylogroups. On average, *P*. *viridiflava* isolates harboured roughly four T3SS effectors, as compared to an average of about 30 T3SS effectors detected in primary *P*. *syringae* phylogroups (Dillon, Almeida, et al., 2019).

### 5.4 Other potential virulence factors

While pathogenicity islands, phase variants, and soft rot capability are the main contributors to virulence, there are a few other potential virulence factors in the arsenal of *P*. *viridiflava*. The *P*. *syringae* group in general is known for its use of toxins, particularly coronatine, syringomycin, syringopeptin, tabtoxin, and phaseolotoxin, in induction of plant disease (Bender et al., 1999). Although *P*. *viridiflava* isolates in PG7 have not been shown to produce toxins, isolates in PG8 all produced a toxin inhibiting the fungus *Geotricum candidum* in an in vitro bioassay (Berge et al., 2014). Because *P*. *viridiflava* probably does not produce syringomycin, it is possible that the toxicity could be a product of an antimycotic, ecomycin, that was previously identified as a toxin produced by *P*. *viridiflava* (Berge et al., 2014; Miller et al., 1998). The production of ecomycin may serve as a virulence factor by means of eliminating fungal competitors.

Another potential virulence factor is ice nucleation activity (INA). This is a general characteristic that spans across the phylogroups of the *P*. *syringae* complex at varied intensities. Currently, only isolates belonging to PG7 have been shown to have INA; in one study, 33% of PG7 isolates tested and none of the PG8 isolates were ice nucleation active (Berge et al., 2014). However, for the isolates that do exhibit INA, this may be an important virulence factor in creating frost damage and thus creating a wound in a plant host for bacterial infection. This was exemplified in a study where kiwifruit plants were infected with an ice nucleation active strain of *P*. *viridiflava* and when cooled down to −3 °C the presence of the bacteria increased frost sensitivity in accordance with bacterial concentration (Varvaro & Fabi, 1992). Although not as prominent as other virulence factors, INA ability may enhance the pathogenic potential of some strains of *P*. *viridiflava*.

## 6 SCOPE FOR FUTURE RESEARCH

*P. viridiflava* is a relatively genetically diverse species that can act as an endophyte, epiphyte, saprophyte, and pathogen with the capacity to acquire new traits through HGT. As a pathogen, *P*. *viridiflava* has a wide host range and has been responsible for several disease outbreaks since the start of the century. An introduction to a novel, susceptible host plant or an unexpected evolutionary shift in the *P*. *viridiflava* population, perhaps a genetic recombination with pathogenic *P*. *viridiflava* from a crop and an environmental *P*. *viridiflava* isolate, could be responsible for the next sweeping plant epidemic.

Based on the recent research on *P*. *viridiflava*, there are several needed areas of research focus. First, *P*. *viridiflava* has been severely under‐sampled compared to other members of the *P*. *syringae* species complex. The pan‐genome of *P*. *syringae* as a species complex needs to be studied more extensively, that is, isolating more environmental isolates that include *P*. *viridiflava*‐grouping strains from PG7 and PG8. Because *P*. *viridiflava*‐related sequences have been isolated from environmental samples (rain, irrigation, snowpack, etc.) in addition to plants, there is probably a plethora of untapped diversity within this species waiting to be explored. Isolating more *P*. *viridiflava* will help uncover the evolutionary potential of this species.

Second, future research should also include extensive virulence/pathogenicity testing on a broad range of hosts under varied environmental conditions. Although there is already a long list of hosts in which *P*. *viridiflava* can cause disease, it will be necessary to test pathogen capabilities on economically important hosts with *P*. *viridiflava* strains isolated from both agricultural and environmental contexts. Extensive host range testing will help in the understanding and prediction of potential host jumps or even potential epidemics/outbreaks. Further host range testing under variable climate conditions (temperature, humidity, etc.) will also assist in these predictions. The host range testing of *P*. *syringae* isolates in Morris et al. (2019) is an example that needs to be followed for a wide range of PG7 and PG8 *P*. *viridiflava* isolates.

Third, the techniques used to determine diversity within *P*. *viridiflava* (and *P*. *syringae* more generally) should shift away from MLSA, 16S rRNA gene sequences, and rep‐PCR and move toward whole‐genome sequencing. As whole‐genome sequencing becomes more accessible and affordable, whole genomes of *P*. *viridiflava* and other members of the *P*. *syringae* phylogroups should be used in taxonomic and phylogenetic characterization. This is important because there are features of the whole genome that will assist in effective and meaningful characterization of *P*. *viridiflava* isolates that are not captured by housekeeping or 16S rRNA genes such as virulence factors, genome level variations, and more. Results reported by Gomila et al. (2017) and Dillon, Thakur, et al. (2019) are examples of whole‐genome sequencing studies that resulted in new knowledge of *P*. *viridiflava* characteristics that may not have been obvious with MLSA or single‐gene analysis.

To prevent and control plant disease, it is necessary to be able to detect the pathogen. Fortunately, there have been advances in detection methods of *P*. *viridiflava* in recent years. PCR primers for lipoprotein and monooxygenase genes, which are present in the majority of *P*. *viridiflava* strains regardless of PAI type, were created for species‐specific detection (Bartoli et al., 2014). Primers for the lipoprotein and monooxygenase genes in *P*. *viridiflava* (Bartoli et al., 2014) have been used in multiplex PCR with primers for the lipodepsipeptide toxin gene (Sorensen et al., 1998) present most commonly in *P*. *syringae* sensu strico for the detection and diagnosis of pathogens causing bacterial stem blight of alfalfa (Lipps et al., 2019). Currently, there are no highly effective methods for management of *P*. *viridiflava* diseases. Generally, elimination or reduction of pathogen inoculum is recommended for diseases caused by the *P*. *syringae* species complex (Lamichhane et al., 2015). For *P*. *viridiflava*, the recent discovery of irrigation water, streams/rivers, snowpack, and epilithic biofilms serving as inoculum sources should shape the practices for eliminating or reducing pathogen inoculum. There have been some successes using *Bacillus* as a biocontrol in vitro, as well as some promise in using copper compounds to control epiphytic populations (Balestra & Bovo, 2003; Orel, 2020). Additionally, further exploration of the mechanisms behind the nonpathogenic transparent phase variants of *P*. *viridiflava* could pave the way for developing control strategies based on increasing the occurrence of these variants (Bartoli et al., 2015).

As far as achieving disease resistance, the use of translational taxonomy and the application of basic taxonomic research to advance knowledge regarding disease control will be crucial in the case of *P*. *viridiflava* due to its muddled relationship to the *P*. *syringae* complex. Classifying, naming, and identifying isolates of *P*. *viridiflava* based on relevant characteristics will enhance the ability of researchers to develop resistant plants. For example, current knowledge of pectolytic capability, PAI type diversity, and effector and (a)virulence gene repertoires specific to *P*. *viridiflava* will help accelerate research on *P*. *viridiflava‐*specific avenues of disease resistance. At present, there are no cases of plants bred or engineered specifically for resistance of diseases caused by *P*. *viridiflava*.

## Data Availability

Data sharing is not applicable to this article as no new data were created or analysed.
